# Advancing Cycling Safety: On-Bike Alert System Utilizing Multi-Layer Radar Point Cloud Clustering for Coarse Object Classification

**DOI:** 10.3390/s24103094

**Published:** 2024-05-13

**Authors:** Asma Omri, Noureddine Benothman, Sofiane Sayahi, Fethi Tlili, Ferdaous Chaabane, Hichem Besbes

**Affiliations:** 1 COSIM Lab, Higher School of Communication of Tunis, University of Carthage, Ariana 2083, Tunisia; ferdaous.chaabene@supcom.tn (F.C.); hichem.besbes@supcom.tn (H.B.); 2 Innovation Department, ACTIA Engineering Services, Ariana 2083, Tunisia; sofiane.sayahi@actia-engineering.tn; 3 IP Department, ACTIA Engineering Services, Ariana 2083, Tunisia; noureddine.benothman@actia-engineering.tn; 4 GRESCOM Lab, Higher School of Communication of Tunis, University of Carthage, Ariana 2083, Tunisia; fethi.tlili@supcom.tn

**Keywords:** multi-layer clustering, coarse classification, point cloud, micromobility dataset, warning system, cyclist safety

## Abstract

Cyclists are considered to be vulnerable road users (VRUs) and need protection from potential collisions with cars and other vehicles induced by unsafe driving, dangerous road conditions, or weak cycling infrastructure. Integrating mmWave radars into cycling safety measures presents an efficient solution to this problem given their compact size, low power consumption, and low cost compared to other sensors. This paper introduces an mmWave radar-based bike safety system designed to offer real-time alerts to cyclists. The system consists of a low-power radar sensor affixed to the bicycle, connected to a micro-controller, and delivering a preliminary classification of detected obstacles. An efficient two-level clustering based on the accumulation of radar point clouds from multiple frames with a temporal projection from previous frames into the current frame is proposed. The clustering is followed by a coarse classification algorithm in which we use relevant features extracted from the resulting clusters. An annotated RadBike dataset composed of radar point cloud data synchronized with RGB camera images is developed to evaluate our system. The two-level clustering outperforms the DBSCAN algorithm, achieving a v-measure score of 0.91, compared to 0.88 with classical DBSCAN. Different classifiers, including decision trees, random forests, support vector machines (SVMs), and AdaBoost, have been assessed, with an overall accuracy of 87% for the three main object classes: four-wheeled, two-wheeled, and others. The system has the ability to improve rider safety on the road and substantially reduce the frequency of incidents involving cyclists.

## 1. Introduction

As vulnerable road users, cyclists are more likely to suffer severe or even fatal injuries in traffic accidents. According to the National Highway Road Safety Administration, there were 966 bicycle deaths in US road crashes in 2021 [1]. As cycling becomes a more and more popular form of transportation, innovative methods are needed to improve road safety for VRUs and, in particular, cyclists. Currently, the inadequacy of the availability, efficiency, and effectiveness of safety devices and accessories for cyclists is conspicuous. For example, traditional rear view visors, despite being commonly used, do not come as standard features on bicycles but are typically added afterwards with uncertain quality. Similarly, conventional bicycle mirrors exhibit constraints like significant blind spots and challenges in determining speed and distance from vehicles approaching from the rear, especially when cyclists are leaning forward to reduce wind resistance. Notably, bicycle mirrors are prone to damage and introduce additional risks to cyclists in the event of an accident.

The installation of a radar at the back of a bicycle significantly boosts safety by supplying cyclists with real-time recognition of their environment. Unlike standard rear-view mirrors, radar technologies provide inclusive environment perception without necessitating that cyclists divert their focus from the path in front. Even in situations with limited vision, the mmWave radar system can detect and track objects with high accuracy. It has been efficient in tracking and detecting objects in real time.

Cost-effective and efficient safety systems for bicycles are needed considering their unique characteristics and infrastructure limitations. An on-board early warning system using mmWave radar and ultrasonic sensors is developed in [2] to detect risks during lateral maneuvers carried out by the cyclist. The authors of [3] propose to transpose an Intelligent Transportation System (ITS) used for cars into a bike. They use four radars, providing full 360° coverage, which is a power-harvesting system for a bike since it uses a battery as a power source. Another approach [4] consists of the fusion of a camera and radar as a solution to the power-saving problem in e-bikes. However, further development is needed to optimize the sensor fusion parameters and improve the human–machine interface to enable real-time warnings. A miniaturized system [5] detects and tracks motorized vehicles passed by a bicycle rider while maintaining minimum power consumption. The authors of [6] propose the development of a 24 GHz FMCW front-end radar for Pedal Electric Cycles (Pedelec) configured with several antennas to identify possible threats based on a range-velocity analysis and multi-target object tracker with a Kalman Filter.

These research works mainly concentrate on designing and evaluating radar systems uniquely intended for bicycles. Their goal is to address the special difficulties faced by cyclists on the road because of their smaller size relatively to other vehicles. One aspect regularly covered in these papers is the design of and the detection optimization and tracking algorithms for mmWave radar systems. Researchers examine techniques for accurately identifying and tracking nearby objects, like vehicles, pedestrians, and other riders, by reducing false positives or false situational threats. Only one study [4] has addressed the problem of object classification with reference to a camera to acquire further object information.

Placing a radar at the back of a bicycle can significantly enhance safety by increasing visibility and reducing the risk of accidents. For this reason, a low-complexity radar-based system is proposed, consisting of the following:

1. An mmWave radar-based system operating at 77 GHz connected to a micro-controller, communicating with the cyclist’s mobile device via Bluetooth and providing preliminary classification results of nearby obstacles.
2. A RadBike dataset: an annotated dataset of radar point cloud data synchronized with an RGB camera sensor designed for micromobility applications.
3. An effective two-level clustering approach consisting of first-level velocity and a second-level position clustering based on temporal projections of initial clusters for a fixed number of frames.
4. A coarse classification approach based on the temporal and spatial features of the resulting clusters with high accuracy in classifying nearby objects proposed for the main classes: four-wheeled, two-wheeled, and others.

This paper is organized as follows. Section 2 provides different approaches related to the detection and classification of road users with mmWave radars. Section 3 provides an overview of the proposed system and the classification diagram. Section 4 presents our collected dataset, RadBike, and the evaluation dataset, View of Delft (VoD). Section 5 describes the projection method and the two-level clustering approach, as well as the hyperparameter tuning and performance evaluation of the clustering approach. Section 6 presents the relevant features extracted from the final clusters and the classification results. Finally, Section 7 concludes the paper and outlines ideas for future work in this area.

## 2. Related Work

Object classification in the field of micromobility remains a relatively new research area. Indeed, there are few papers that have dealt with radar systems for bicycles to enhance cyclists’ safety on the road.

### 2.1. Deep Learning Based Classification

The radar data classification task depends on the radar data level, whether it involves point clouds, low-level radar signals, or a combination of both. Deep learning techniques have been widely used in mmWave radar classification. The authors of [7] have used the PointPillars approach proposed in [8] to extend the application of this detector from Lidar point cloud detection to radar point cloud detection. The detector uses an encoder based on PointNets to represent point clouds in vertical columns.

The low-level radar signal-based classification has been proven to be more effective in detecting and classifying vehicles with large dimensions compared to conventional approaches such as density-based spatial clustering [9] and support vector machines [10]. The work in [11] introduces a deep neural network based on convolutional recurrent units that captures the dynamics of moving objects using range–Doppler heat maps as input. Another approach [12] focuses on utilizing one-dimensional convolutional neural network (1D-CNN) models for the classification of multiple targets in complex radar environments. Both micro-Doppler and micro-range spectrograms are created for multiple objects concurrently, and 1D time sequence features are extracted for classification using a 1D convolutional neural network (CNN).

Range–azimuth heatmaps have been used in [13] to simultaneously detect and classify objects using the YOLO (You Only Look Once) deep learning model with pre-processed automotive radar signals. The work proposed in [14] combines an FMCW radar, a YOLOv7 model, and a Pix2Pix architecture to enhance object classification accuracy. The authors improved the YOLOv7-X model by incorporating it into YOLOv7-PM to clean up the heatmaps before feeding them into the detector. This enhancement leads to surpassing the performance of the base YOLOv7-X model by an improved mAP score of 91.82%.

A combination of both point cloud and low-level data has also been a success in multi-class detection and classification. The work in [15] combines object positions extracted from target-level data and velocity distributions from low-level radar data. A convolutional neural network (CNN) is then employed to classify the objects generated by clustering based on class scores, positions, and velocities. The authors of [16] developed a lab-scale radar test bed using Texas Instrument’s mmWave radar and generated a large raw radar dataset. They used raw data to extract target micro-Doppler signatures and feed them into a VGG16 deep learning classifier.

Despite the impressive performance of deep learning approaches in radar object classification, there remains a challenge due to their high computational complexity. Implementing such systems on a bicycle, for instance, would be impractical given the computational demands.

### 2.2. Machine Learning-Based Classification

To address the issue of the high complexity of DL approaches, researchers are increasingly exploring machine learning approaches that leverage efficiency and resource optimization. These ML approaches make radar object classification systems more suitable for real-time applications and low-demand computational systems.

The authors of [17] propose a point cloud features-based kernel SVM algorithm for human–vehicle classification. They introduce a new feature vector with eleven dimensions to describe the shape, velocity, and intensity of point clouds. The use of those features as input to the SVM classifier demonstrates high accuracy in the classification task. Another work in [18] consists of a low-cost 24 GHz FMCW radar based on features extracted from the Doppler FFT and range FFT for accurate target recognition and classification.

Unsupervised learning methods like clustering [19] were introduced for point cloud-based classification. Further processing is carried out by extracting meaningful features from clusters, which will be used in the classification task. The success of the classification task primarily depends on the success of the clustering process. In [20], conventional clustering is enhanced into two-stage clustering. In the first stage, early clusters are formed, and the second stage integrates these clusters while extracting additional characteristics, such as object centers and real non-radial velocities, to combine pre-clustered data points into larger clusters. The authors of [21] employ an inter-frame DBSCAN clustering approach with a modified algorithm using Doppler features and a multi-frame merging process to improve classification accuracy. In contrast, a limited number of detections can impact classification accuracy. The authors of [22] adopted a different method permitting accuracy enhancement by extracting different information from point-cloud data at the detection and target levels. They propose a vector with thirteen extracted features for target-level classification, determining optimal classification approaches based on testing accuracy and runtime evaluation.

### 2.3. Hybrid Classification

In addition to deep learning and supervised and unsupervised learning methods, there have been efforts to develop hybrid methods that combine different techniques for better performance. A novel approach that combines a support vector machine (SVM) and a deep learning model, YOLO, was in introduced in [23] to classify human and vehicle objects. It uses range–angle information from high-resolution automotive radar systems to improve the overall classification performance. Another hybrid technique was proposed in [24] and consists of a combination of convolutional neural networks (CNNs) and support vector machines (SVMs) to improve classification accuracy. The work in [25] proposes a new algorithm, clustered ESPRIT, for accurate angle and range estimation by combining the ESPRIT algorithm with signal clustering. The algorithm is an efficient algorithm and can handle multiple targets with the same ranges or angles. The algorithm was validated by numerical simulations and practical experiments.

## 3. Proposed System

As a key factor, this work must consider the need for the radar system to have low complexity, as it is intended to be installed on bicycles. Keeping the design simple is important since these vehicles have limited space and carrying capacity compared to larger vehicles. The radar needs to be straightforward and easy to integrate while still fulfilling its function of detection at a basic level.

**Figure 1:**
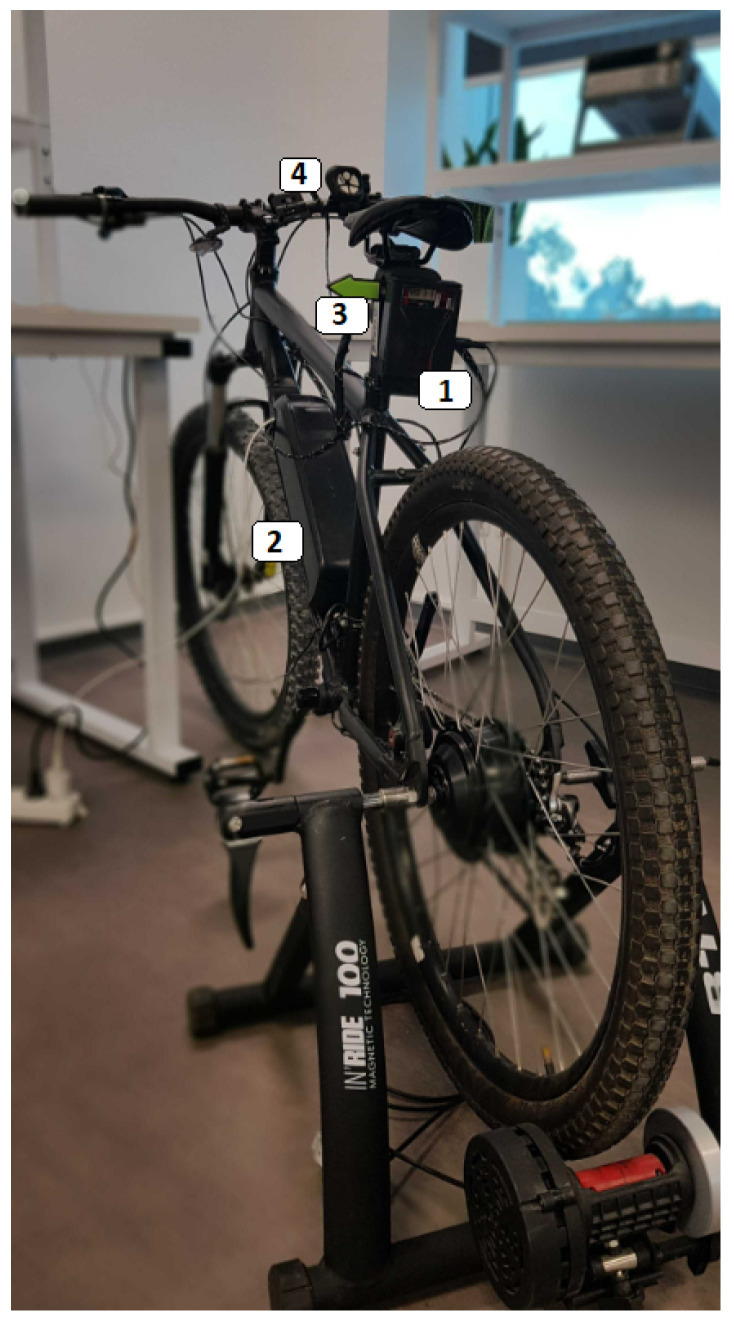
System setup on the bike: (1) TI’s mmWave radar AWR1843BOOST, (2) micro-controller, (3) Mobile device of the cyclist, (4) signal box.

The proposed system in this paper, shown in Figure 1, relates to the system proposed in the patent [26] and enhances protection for bicycle riders by employing an mmWave radar to identify moving objects. When a cyclist requests help to carry out risky maneuvers like crossing several lanes, a roundabout, or an intersection in a circular direction, the radar is activated, and the request of the cyclist is displayed on the signal box (4).

**Figure 2:**
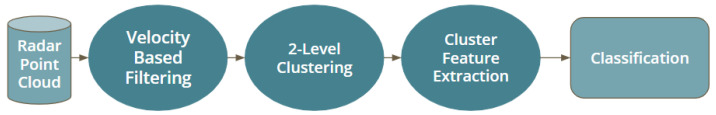
Classification diagram.

The system utilizes a TI’s mmWave radar AWR1843BOOST (1) operating at 77 GHz and processing radar point cloud information, including the position (*x*, *y*), relative radial velocity, and azimuth of objects. The radar is mounted on the back of the bicycle and connected to a micro-controller (2) that interfaces with the sensor via UART to retrieve the data from the radar and run further processing for the classification task presented in Figure 2. The system is considered a low-energy system that is powered via two power banks: one for the radar and one for the micro-controller.

The classification results are sent to the cyclist’s mobile device (3) via Bluetooth to provide alerts related to risky obstacles coming from behind the bicycle. The classification workflow is described as follows:

1. Radar detection filtering: initially, the radar detection data are filtered based on velocity. This step ensures that only relevant moving objects are considered for further analysis.
2. Two-level clustering algorithm: this is applied to the filtered radar data. The resulting clusters contain groups of objects with similar characteristics.
3. Feature extraction: Relevant features are extracted from the clustered data. These features serve as input for the subsequent coarse classification algorithm.
4. Coarse classification into three main classes: Our coarse classification algorithm segments the clusters into three primary classes:
  - Four-wheeled class: Comprising trucks and cars. These large, high-speed objects pose significant risks to cyclists due to their dimensions.
  - Two-wheeled class: Consisting of motorcycles and bicycles. These modes of transport share the road with cyclists and exhibit similar mobility patterns. While still relevant, they pose comparatively lower risks.
  - Others class: Encompassing pedestrians and any remaining objects not falling into the above categories. This includes people walking into the back of the bicycle, which does not pose a major risk to the cyclist.

It is worthy to note that the decision to consolidate classes specifically grouping trucks and cars, as well as bikes and motors, is grounded in practical considerations. This choice is argued by the need to design an efficient system for rear-end collision prevention. By merging similar vehicle types, the classification process is streamlined, ensuring practical feasibility during its implementation. The resulting simplicity facilitates its seamless integration with existing radar systems. Rear-end collisions involving bicycles and various vehicles (trucks, buses, cars, and motorcycles) are common. The radar installation prioritizes rear detection capabilities, where such collisions frequently occur. The amalgamation of trucks and cars, as well as bikes and motors, is driven by pragmatic considerations. A lightweight approach is maintained to uphold system effectiveness while minimizing complexity. Limiting the classes to three ensures practicality without compromising hazard recognition. Moreover, the application not only classifies vehicles but also triggers alarms as vehicles approach, enhancing safety awareness and providing critical information such as speed, direction, and time to contact.

## 4. Datasets

### 4.1. Evaluation Dataset: VoD

The view for the Delft VoD [7] dataset consists of a collection of synchronized 4D radar point clouds along with images, Lidar data, and odometry data, each sampled at a frequency of 13 frames per second and captured from various viewpoints in the city of Delft in the Netherlands. The radar point clouds have a range resolution of 0.2 m, a velocity resolution of 0.1 m/s, and an angular resolution of 1.5° for both azimuth and elevation. The dataset provides a diverse range of urban scenes, including streets, buildings, pedestrians, and vehicles, collected for a maximum range of 100 m. For the purpose of these experiments, our radar point cloud data have been used with this information [*x*, *y*, RCS, $v_{r}$, $v_{r_{compensated}}$, time], where *x* and *y* are the coordinates of the detection location in meters, RCS is the radar cross section, which is the reflectivity of the object by the radar, $v_{r}$ is the relative velocity returned by the radar, $v_{r_{compensated}}$ is the compensated relative velocity, and time is the number of radar frames.

Ground truth images were used to annotate the radar detection for the clustering task. The elevation dimensions were not included since the proposed system used a 3D radar. The annotations are object-level, which includes 13 different classes, and provided in the bounding box format extracted from the camera sensors within 50 m. Hundreds of frames were annotated to evaluate the two-level clustering and classification algorithms. The new annotations include the three main classes: four-wheeled, two-wheeled, and others.

### 4.2. Proposed RadBike Dataset

#### 4.2.1. Dataset Description

Radbike includes a one-hour length of synchronized radar frames and RGB camera images that show various traffic scenarios in the urban area of Ariana, Tunisia. A total of 3000 of the 36,000 frames are annotated, each one at a rate of 10 frames per second. The annotations give precise position information for the objects of interest using the YOLOv5 2D bounding box format. The annotated frames contain mainly three classes, including four-wheeled, two-wheeled, and others, providing a comprehensive representation of the vehicle entities in the environment. The radar data are in the format of point cloud detections, and each detection data point comes with the following information: [$frame_{index}$, timestamp, $point_{id}$, *x*, *y*, range, velocity, angle, intensity]. The dataset flexibility for testing and assessing machine learning models is increased by the inclusion of sequences captured in a variety of setup conditions, including with a fixed radar, a moving radar on a car, and a moving radar on a bike.

#### 4.2.2. Data Acquisition System

**Figure 3:**
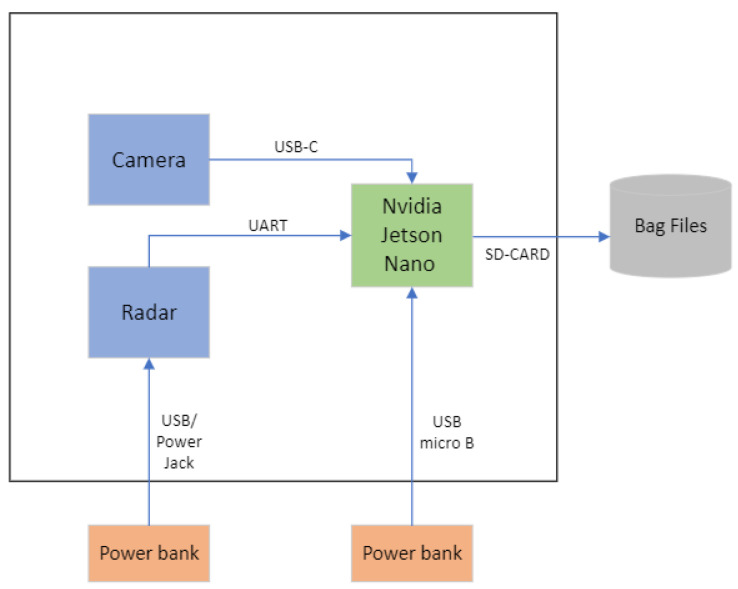
Data acquisition system.

The data acquisition system is composed of TI’s radar sensor AWR1843BOOST and the Intel Realsense Camera D435. The two sensors are connected to the NVIDIA Jetson Nano board, as shown in Figure 3. The system is powered by two power banks, one for the radar and the other for the NVIDIA Jetson Nano board, permitting the necessary mobility to install the system on a bike. The data extracted from the radar and camera sensors will be stored on an SD card to be processed later for the synchronization task. We configured the radar and camera as shown respectively in Table 1 and Table 2. The radar is able to detect obstacles from up to 100 m away, according to the evaluation dataset, VoD. The difference between the two configurations is the resolutions for the range, the velocity, and the azimuth. The RadBike dataset employs a low-resolution radar, whereas the evaluation dataset, VoD, employs a high-resolution radar.

#### 4.2.3. Offline Software Synchronization

**Figure 4:**
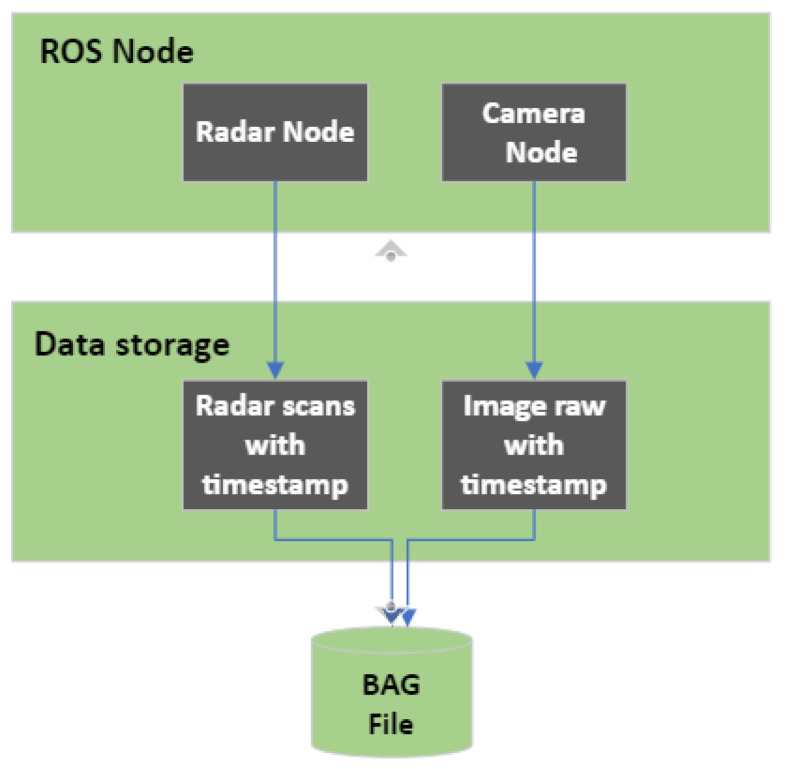
Radar and camera nodes in the ROS framework.

The two sensors run at different frequencies: 10 FPS for the radar and 25 FPS for the camera. Thus, the accurate synchronization of data between the radar and camera is critical for our system. It ensures that the data from both sensors align with the same timing, enabling a meaningful analysis. To handle this communication, a robot operating system (ROS), the middleware system, was used, providing a unified timestamping mechanism. The radar node and camera node were developed separately, as depicted in Figure 4. The radar node reads radar data and publishes it as ROS messages with appropriate timestamps. The camera node captures RGB and depth images and publishes them as ROS messages with corresponding timestamps. The radar frame is set as a reference for synchronization since it has a lower sampling frequency compared to the camera. An algorithm is developed to match the timestamps between the two sensors. Thus, matching pairs of frames were identified.

#### 4.2.4. Cluster Level Annotation

Since the input of the classification algorithm is the clusters along with their deduced features, hundreds of frames were re-annotated in the evaluation dataset as well as the proposed dataset in the cluster-level annotations. The new annotations are provided for five successive frames, following the work that was carried out in [7]. The resulting format of the radar point cloud information is [$v_{c}$, $d_{x_{c}}$, $d_{y_{c}}$, $d_{c}$, $rcs_{eq}$, $rcs_{std}$, $class_{id}$]. Around 750 clusters for multiple frames of VoD and RadBike were annotated. These annotated clusters will be used as the training and testing sets in the classification task.

## 5. The Two-Level Clustering

### 5.1. Methodology

In a single frame, various objects are represented by several point clouds, particularly those with smaller dimensions. This requirement involves considering a specific number of past detections for clustering purposes. The accumulation of frames leads to a sparsity of points along the x-dimension, particularly when dealing with large-dimensional objects such as cars and trucks. This sparsity, in turn, gives rise to the appearance of multiple clusters. To tackle this challenge effectively, a method is proposed to reduce the dimensionality of the clusters by compacting them along the x-axis. Consequently, these adjustments minimize the length of the clusters over multiple frames, Δt.

Recall that the radar provides, for each detected point, an estimation of its range, $r_{i}$, radial velocity, $v_{r_{i}}$, and azimuth angle, $\theta_{i}$, and the relationship between the radial velocity and velocities along x and y axes can be approximated by

(1) $$v_{r_{i}}=\cos\theta v_{x_{i}}+\sin\theta v_{y_{i}}.$$

At low azimuth angles, the velocity along the x-axis can be approximated as follows:

(2) $$v_{x_{i}}=\frac{v_{r_{i}}}{cos(\theta_{i})}.$$

The approximation for the projection of point cloud *i* from the previous time step, t−Δt, to the current time, *t*, along the x-dimension can be expressed as follows:

(3) $${\hat{x}}_{i}\left(t\right)=x_{i}\left(t-\Delta t\right)+\Delta t\;\frac{v_{r_{i}}}{cos(\theta_{i})}.$$

**Figure 5:**
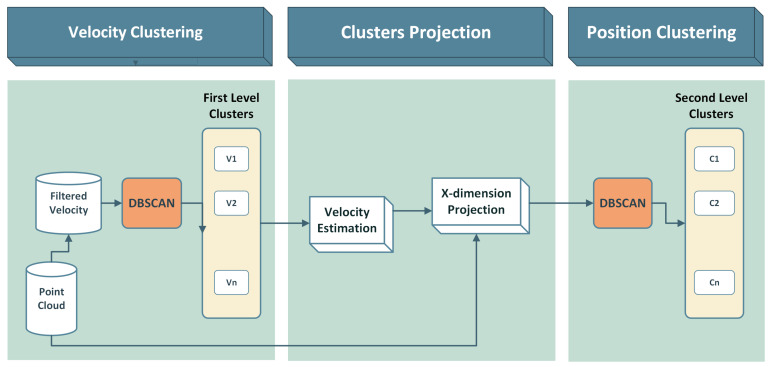
Two-level clustering diagram.

Following the accumulation of a point cloud through projection, our proposed clustering methodology, as illustrated in Figure 5, adopts a two-level approach. This method is based on the state-of-the-art Density-Based Spatial Clustering of Applications with Noise (DBSCAN) algorithm, as detailed in [9]. Two separate levels define the clustering process: the first level is velocity-dependent, while the second level is position-dependent.

#### 5.1.1. First Level: Velocity-Based Clustering

**Figure 6:**
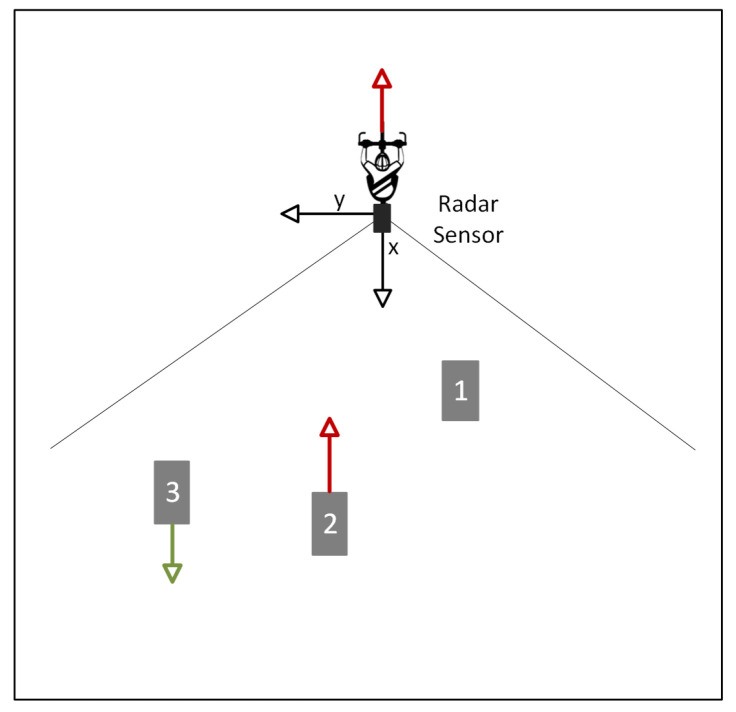
An illustration of objects’ motion in the radar field of view (FoV): object 1 is static; object 2 is moving towards the bike; and object 3 is moving away from the bike.

In the optics of our application, only dynamic objects moving toward the bike are considered, and static objects are filtered out. In this process, a crucial consideration arises from the dynamic nature of the observed scene. When the bike is in motion, as depicted in Figure 6, static objects perceived from behind may create an illusion of movement away from the observer, resulting in a positive radial velocity. To address this issue, one would selectively focus on a point cloud with a negative velocity, particularly when the bike is moving forward. By doing so, points associated with static or non-relevant motion are filtered out, as objects in motion will predominantly exhibit a negative radial velocity during the forward movement of the bike. Consequently, those points are identified as core points and integrated into the clustering process.

#### 5.1.2. Second Level: Position-Based Clustering

The second level of clustering takes as input the results obtained from the first level. For each cluster, $v_{i}$, that shares a similar velocity profile, a set of sub-clusters to be identified as individual objects was explored. In this process, the primary criterion is the object position, which serves as the main parameter for the second DBSCAN algorithm.

The outcome of second-level clustering is a refined set of clusters denoted by $\left(c_{1},c_{2},\cdot \cdot \cdot ,c_{N_{c}}\right)\;\in \;C$, where $N_{c}$ represents the number of clusters. These clusters now offer a more accurate representation of the spatial distribution of the objects, rendering them suitable for subsequent classification tasks. Each cluster encompasses a more homogeneous set of data points in terms of spatial proximity, enhancing its representational quality.

### 5.2. HyperParameter Tuning

The success of the two-level clustering approach depends primarily on the chosen parameters for the DBSCAN algorithm for the levels of the proposed approach. These parameters are the minimal number of neighbors, $N_{min}$, and the epsilon threshold, ε, for a neighborhood search radius needed to consider a point as a core point. However, the optimization of these parameters requires having pointwise-level annotations of the radar point cloud, which will serve to distinguish between the ground truth clusters and the resulting clusters. But this is not the case for RadBike and VoD, which contain bounding box annotations from the camera’s view. Therefore, manual annotations for hundreds of frames were necessary in order to identify the point clouds related to the objects and those considered a clutter environment. The annotations were carried out on the dynamic objects obtained from the velocity-filtering phase. The resulting data comprise the radar point cloud detection along with the $cluster_{id}$, given in the format [*x*, *y*, RCS, $v_{r}$, $v_{r_{compensated}}$, time, $cluster_{id}$].

For the optimization task, the hyperparameters of the first-level clustering were fixed to identify big groups of points that have similar velocities. The chosen parameters were $\varepsilon_{1}=0.5$ and $N_{min_{1}}=3$. Secondly, to optimize the hyperparameters of the second-level clustering, the v-measure score [27] was computed, which is based on two key metrics: homogeneity and completeness. Homogeneity reaches its peak when a resulting cluster contains only data points from a single true cluster. Completeness, on the other hand, aims to combine every point from a certain ground truth cluster into a single cluster. Expressions for homogeneity and completeness are given in [27], as follows:

(4) $$h=\left\{\begin{array}{cc} 1 & \;\mathrm{if}\;H(C,K)=0\\ 1-\frac{H(C|K)}{H(C)} & \;\mathrm{else}\; \end{array}\right.$$

(5) $$c=\left\{\begin{array}{cc} 1 & \;\mathrm{if}\;H(K,C)=0\\ 1-\frac{H(K|C)}{H(K)} & \;\mathrm{else}\; \end{array}\right.$$

where

(6) $$\begin{array}{cc} H(C|K) & =-\sum_{k=1}^{\left|K\right|}\sum_{c=1}^{\left|C\right|}\frac{a_{ck}}{N}\log\frac{a_{ck}}{\sum_{c=1}^{\left|C\right|}a_{ck}}\\ H(C) & =-\sum_{c=1}^{\left|C\right|}\frac{\sum_{k=1}^{\left|K\right|}a_{ck}}{n}\log\frac{\sum_{k=1}^{\left|K\right|}a_{ck}}{n} \end{array}$$

(7) $$\begin{array}{cc} H(K|C) & =-\sum_{c=1}^{\left|C\right|}\sum_{k=1}^{\left|K\right|}\frac{a_{ck}}{N}\log\frac{a_{ck}}{\sum_{k=1}^{\left|K\right|}a_{ck}}\\ H(K) & =-\sum_{k=1}^{\left|K\right|}\frac{\sum_{c=1}^{\left|C\right|}a_{ck}}{n}\log\frac{\sum_{c=1}^{\left|C\right|}a_{ck}}{n} \end{array}$$

$a_{ck}$ is the number of point clouds that are members of class *c* and elements of cluster *k*. The homogeneity and completeness clustering criteria are combined to provide an aggregated score, which is the V-measure [27], given by (8). β is used for weighting between the two metrics. In this equation, completeness is given a higher weight if β is more than 1, and homogeneity is given a higher weight if β is less than 1. To maintain an equal balance between the two metrics, a value of 1 is chosen; thus, the v-measure becomes

(8) $$V=\frac{h.c}{h+c}.$$

The clustering evaluation metrics are computed for different combinations of hyperparameters of the second-level clustering, as shown in Table 3. The selection was based mainly on the v-measure, which gave the best result of 0.913 for $N_{min_{2}}=3$ and $\varepsilon_{2}=0.7$. The highest level of homogeneity was given for $N_{min_{2}}=2$ and $N_{min_{2}}=4$ and for the same value of epsilon. However, the completeness was much lower compared to the results given for $N_{min_{2}}=3$, which further justifies the resulting compromise between homogeneity and completeness in our choice.

**Figure 7:**
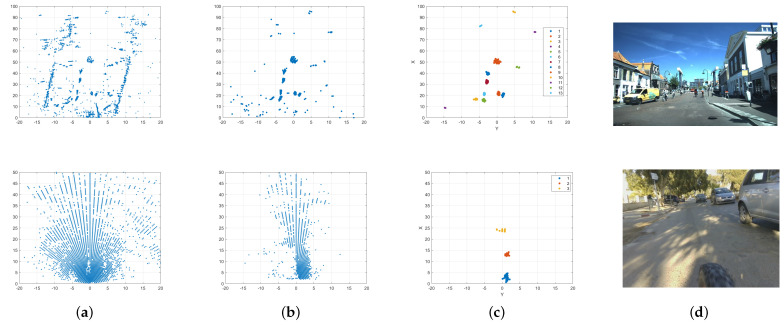
Two−level clustering results for two scenes: **one from VoD with a high-resolution radar and one from RadBike with a low-resolution radar**. (**a**) represents sparse radar point clouds before any processing. (**b**) represents the radar point clouds after velocity filtering. (**c**) shows the results of the 2-level clustering approach. (**d**) represents the ground truth image from the camera for the two scenes.

Figure 7 illustrates the results for each phase of our clustering method for the chosen optimized parameters. For both scenes in VoD and RadBike, the two-level clustering was shown to be effective in detecting objects from high-resolution as well as low-resolution point cloud data.

### 5.3. Performance Evaluation

#### 5.3.1. Performance Comparison

To assess the performance of this clustering approach, a comparative evaluation, specifically of the DBSCAN (Density-Based Spatial Clustering of Applications with Noise) algorithm, was chosen.

**Figure 8:**
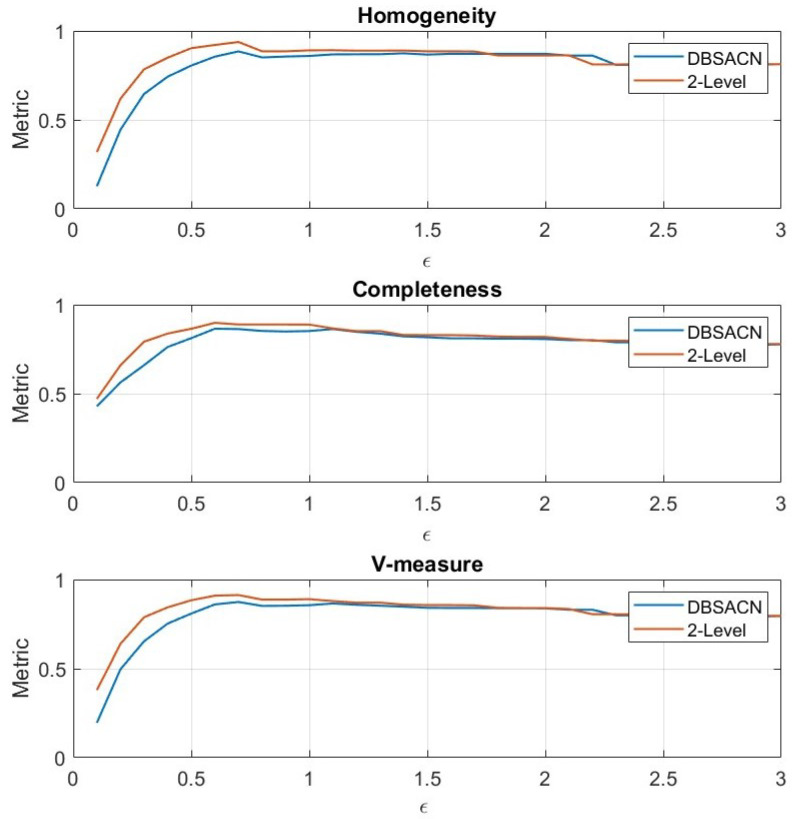
Performance of the two-level clustering approach and DBSCAN algorithm over the hyperparameter ε for each performance evaluation metric.

Homogeneity, completeness, and v-measures were computed over ε for both the two-level approach and the classical DBSCAN algorithm using the selected hyperparameters, $\varepsilon_{2}=0.7$ and $N_{min_{2}}=3$, obtained from the optimization process for both methods to provide the same baseline for the comparison. The proposed method outperforms DBSCAN in terms of the three metrics used for values of ε ranging between 0.1 and around 1, as shown in Figure 8. For values of epsilon beyond 1, the performance of the two methods decreased.

A statistical analysis was included to assess the significance of the difference between our two-level approach and DBSCAN. Several metrics were calculated, as shown in Table 4. In addition to the homogeneity, completeness, and v-measure metrics, as explained in the previous subsection, the adjusted Rand index [28], Davies–Bouldin index [29], and silhouette score [30] were computed.

The adjusted Rand index (ARI) is a corrected measure of the Rand index, which is a basic measure of similarity between two clusterings, but it has the disadvantage of being sensitive to chance. The ARI takes into account the fact that some agreement between two clusterings can occur by chance, and it adjusts the Rand index to account for this possibility. It ranges from −1 to 1, where 1 indicates perfect agreement between the two clusterings, 0 indicates a random agreement, and −1 indicates that the two clusterings are completely different.

The Davies–Bouldin Index (DB) measures the average similarity of each cluster to the other cluster most similar to it. It is calculated based on the ratio between the inter-cluster and intra-cluster distances, ranking well-separated clusters with less dispersion. It ranges from 0 to 1. A lower Davies–Bouldin score indicates more compact and well-separated clusters, while a higher score suggests poorer clustering solutions.

The silhouette coefficient, S, measures the cohesion and separation between clusters, permitting us to compare the cluster quality. It ranges from −1 to 1. A high silhouette score indicates good clustering, while a low score suggests poor clustering.

The analysis of the two clustering approaches, the two-level clustering approach and the DBSCAN clustering approach, from the data shown in Table 4 reveals interesting insights into their performance based on key metrics:

- Two-level clustering tends to have slightly higher mean values across most metrics compared to DBSCAN.
- DBSCAN shows a wider range of performance, with both lower min and higher max values.
- Homogeneity (H) and completeness (C): The two-level clustering approach shows slightly higher values for both H and C compared to DBSCAN, indicating that clusters in this approach are more pure and contain all members of a given class. This suggests a better separation of clusters based on class membership.
- The V-measure (V): the V-measure, which combines homogeneity and completeness, is also higher for the two-level clustering approach, indicating a better balance between precision and recall in clustering.
- Adjusted Rand index (ARI): The two-Level clustering approach has a higher AR value, suggesting a higher level of similarity between clusters compared to DBSCAN. This indicates that the clusters formed in the two-level approach are more consistent across different runs or variations.
- Davies–Bouldin Index (DB): the DB index is lower for the two-level clustering approach, indicating better clustering quality in terms of intra-cluster similarity and inter-cluster differences.
- Silhouette (S): the silhouette score is higher for the two-level clustering approach, indicating that the clusters are more well defined and appropriately separated compared to for DBSCAN.

Overall, the two-level clustering approach seems to outperform the DBSCAN approach in terms of cluster quality and consistency across various metrics.

#### 5.3.2. Impact of Clusters’ Projection on the X-Dimension

**Figure 9:**
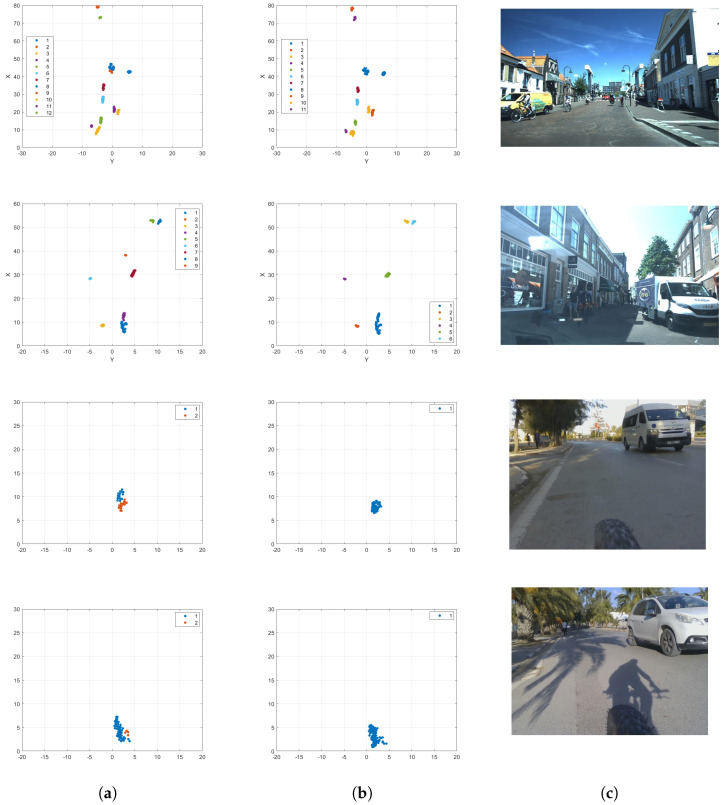
Impact of the x-dimension projection of the clustering for multiple scenes with multiple moving objects, including cars, pedestrians, and two-wheeled objects. (**a**) Clustering results without projection; (**b**) Clustering results with projection; (**c**) Ground truth image.

The effectiveness of the proposed clustering approach is slightly improved by the projection on the x-axis of the clusters over five frames, especially for objects with large dimensions like cars and trucks. The impact of the projection is highlighted in Figure 9 for scenes from both VoD and RadBike. The two first scenes are from the VoD dataset. In the first scene, a car is detected as two clusters, given by cluster 8 and cluster 9, and after the projection, it is detected as one cluster, given by cluster 8. The case is the same for the truck in the second scene; the two clusters 1 and 4 are merged together to form cluster 1.

For the two-wheeled objects presented in the first scene, the dimensions of the clusters have been reduced after the projection. The last two scenes are from our dataset, RadBike, and contain a small bus and a car. For the small bus, it was first clustered into two equivalent clusters, which were merged together into the same cluster after the projection. For the car in the last scene, a few points represented by cluster 2 were detected as a single cluster and were assigned to the same cluster, cluster 1, after the projection.

## 6. Classification Results and Analysis

### 6.1. Feature Extraction

**Figure 10:**
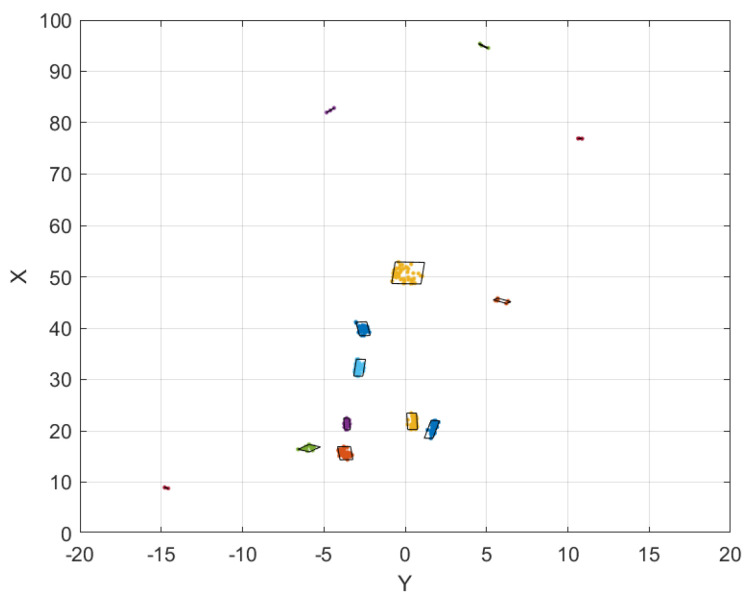
Optimized bounding boxes of the final clusters.

The classification of moving road users depends significantly on the extraction of relevant features from the clusters. These extracted features characterize the object cluster and cover a wide range of information related to its motion, spatial distribution, dimensions, and size. The extracted features are given as follows:

- **The mean velocity**, denoted as $v_{c}$, is the average velocity, $y_{c}$, of the cluster, and it is calculated as the average velocity of all points in the same cluster, given by the following equation:
  (9) $$v_{c_{i}}=\frac{1}{N_{c}}\sum_{j\in C}v_{j}.$$
- **The dimensions**, denoted as ($d_{x_{c}},d_{y_{c}}$), are the dimensions of the estimated bounding box of the cluster represented in Figure 10. The bounding box is an optimization of the rectangular bounding box, covering all the points in the cluster with an estimated orientation of the cluster.
- **The density** represents the spatial distribution of the cluster and is calculated as follows:
  (10) $$d_{c_{i}}=\frac{N_{c}}{S}\;\mathrm{where}\;\;S=d_{x_{c}}\;d_{y_{c}}.$$
- **rcsEq** is the equivalent radar cross section and represents the effective area presented by an object to the radar system. It quantifies the amount of radar energy that is scattered back toward the radar receiver. It takes into account the linear RCS of each point cloud given by $RCS_{j}$ and the signal phase, $\varphi_{j}$, which depends on the operating frequency of the radar, $f_{c}$, and the time delay, $\tau_{j}$, between the transmitted signal and the received signal. It is computed as follows:
  (11) $$\;rcs_{eq_{i}}={\left|\sum_{j}\sqrt{RCS_{j}}e^{j\varphi_{j}}\right|}^{2};$$
  (12) $$\begin{array}{c} \mathrm{where}\;\varphi_{j}=2\pi f_{c}\tau_{j}\;\mathrm{and}\;\tau_{j}=\frac{2R_{j}}{c}. \end{array}$$
- **rcsStd** is the standard deviation of the linear RCS estimated by the radar and refers to the statistical measure of variability in the linear RCS values of a given cluster.

### 6.2. Feature Selection

Feature selection is a primary step in identifying the most relevant features that contribute significantly to the performance of the classification task. Some features often contain redundant information, and considering all of them might lead to unnecessary computational overhead. The minimum redundancy–maximum relevance MRMR [31] algorithm was adopted for feature selection. It combines relevance and redundancy criteria to select an optimal subset of features. The relevance criterion measures the individual importance of each feature, and the redundancy criterion captures the similarity or overlap between features.The calculation of relevance and redundancy is based on mutual information, I(x,y), about two variables, *x* and *y*.

The main idea behind MRMR is to find the best set of features, denoted as *S*, that not only maximizes relevance (represented by $V_{S}$) but also minimizes redundancy (represented by $W_{S}$). This is achieved through a step-by-step process of selecting features, ensuring that their combination provides a rich source of information. It is worth noting that $V_{S}$ and $W_{S}$ are defined using mutual information [31]. The mutual information quotient (MIQ) value is used by the MRMR algorithm to rank features. The feature is referred to as *x*, and the MIQ is given by

(13) $${\mathrm{MIQ}}_{x}=\frac{V_{x}}{W_{x}},$$

where

(14) $$\begin{array}{cc} \; & V_{x}=I\left(x,y\right)\\ \; & W_{x}=\frac{1}{\left|S\right|}\sum_{z\in S}I\left(x,z\right). \end{array}$$

The results of the score are given in Table 5. After studying the correlation between the extracted clusters and features, we were able to choose the most relevant features, which are the mean velocity, the dimensions of the cluster, and the density.

### 6.3. Classification Results

In this work, around 750 samples of clusters were successfully annotated from both the VoD and RadBike datasets. The observed clusters of features within the datasets are significantly shaped by both radars’ resolutions. The resolution difference between both datasets results in different cluster characteristics, which improves the robustness of the classifiers. Four classifiers were assessed, including a decision tree (DT) [32], a random forest (RF) [33], an SVM [10], and AdaBoost [34], to classify objects based on the selected features from the previous subsection.

The results shown in Table 6 confirm that the four classifiers could accurately distinguish between moving object classes, including four-wheeled, two-wheeled, and others. Five-fold cross-validation [35] was adopted to evaluate the performance of the different classifiers. The total sample was split into 70% for the training and validation and 30% for the test. The accuracy for each class and each classifier, as well as the overall accuracy for all classes, was computed. The accuracy is defined by the following:

(15) $$\;\mathrm{Accuracy}\;=\frac{\;\mathrm{True}\;\mathrm{Positive}\;+\;\mathrm{True}\;\mathrm{Negative}\;}{\;\mathrm{Total}\;\mathrm{Sample}\;}.$$

**Figure 11:**
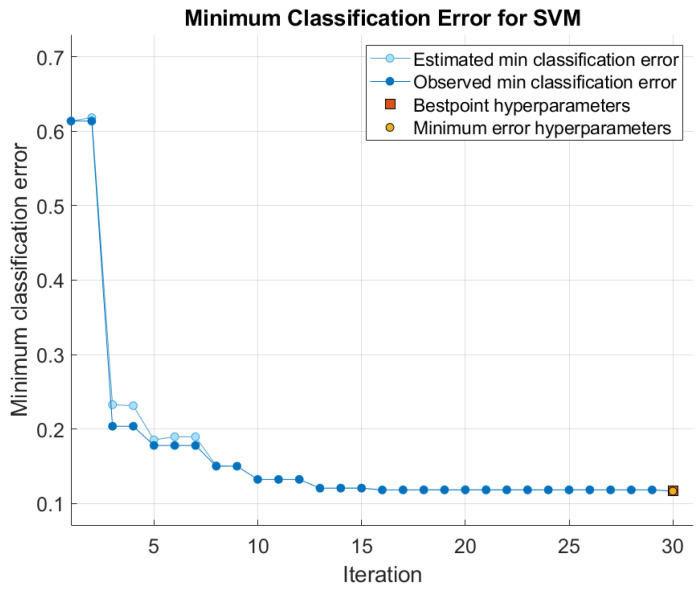
Minimum classification error results over 30 iterations for the optimization of the quadratic SVM kernel.

The SVM classifier was optimized with the Bayesian optimizer [36] using a one-vs-all multi-class method. The minimum classification error (MCE) [37] was computed for 30 sequential iterations, as shown in Figure 11. The optimized hyperparameters of the Gaussian kernel were obtained for the 30th iteration and for an MCE of 0.116.

**Figure 12:**
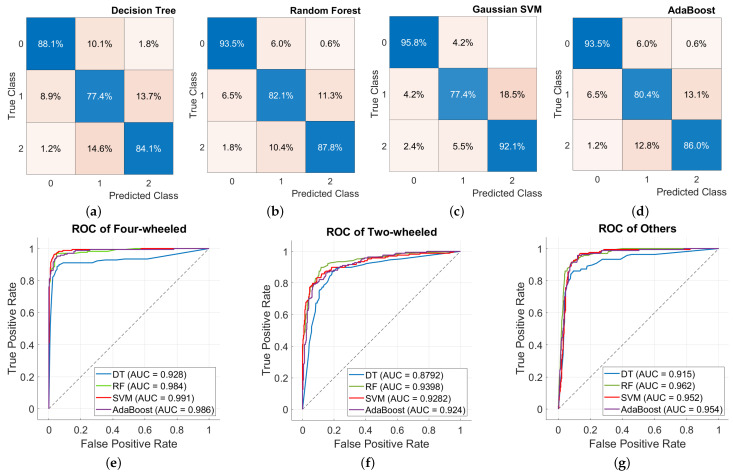
Comparative analysis of the used classifiers in terms of the confusion matrix and receiver operating characteristic (ROC) curves in the validation phase: (**a**–**d**) represent the confusion matrices of each classifier, and (**e**–**g**) represent a comparison between the different classifiers’ ROC curves for each class of objects.

The results of the classification of the proposed method are summarized in Table 6 and Figure 12. The lowest accuracy was obtained for the decision tree, with a value of 83.2%. The highest accuracy was obtained for the Gaussian SVM, with a value of 88.4%. The results of training for the random forest and AdaBoost were obtained with respective values of 87.8% and 86.6%.

In order to prove the effectiveness of the proposed approach, it was compared to the method used in [17], which relies on mmWave radar point clouds for the classification of road users. Noise point clouds were filtered based on a range threshold, and the state-of-the-art DBSCAN was adopted for the clustering task. Eleven features were extracted from the resulting clusters and fed into various kernels of the support vector machine (SVM) to distinguish between two main classes, which are humans and vehicles. To compare the performance of the coarse classification approach, it was necessary to extract the features related to [17] for the clusters resulting from the two-level clustering approach. The accuracy of the different classifiers was computed. The overall difference between our feature set and the feature set from the chosen reference is approximated to be 6% for the validation task.

In the context of implementing a support vector machine (SVM) classifier in a micro-controller, the relationship between the number of features in the test case and the complexity of the SVM implementation is discussed. The complexity of an SVM classifier typically depends on factors like the number of training samples, the type of kernel function used, and, more importantly, the number of features in the data. The computational complexity of an SVM classifier is influenced by the number of features, with a quadratic relationship between the number of features and complexity. This means that as the number of features increases, the computational complexity of implementing an SVM classifier will also increase quadratically.

Assuming that the complexity of the SVM classifier is approximated as $o\left(n^{2}\right)$, with n being the number of features, the proposed feature set is eight times less complex than the compared reference [17]. This confirms the importance of designing efficient and scalable SVM-based classification algorithms.

An encoded class representation was used, as shown below:

- 0: “four-wheeled”;
- 1: “two-wheeled”;
- 2: “others”.

The confusion matrices and receiver operating characteristic (ROC) curves [38] are represented in Figure 12. The confusion matrices display the proportion of accurate and inaccurate predictions for each class. It gives insight into the classes that the model confuses with other classes. The ROC curves show the true positive rate (TPR) against the false positive rate (FPR) for different threshold values. The expressions for the TPR and FPR are shown in (16) and (17). The higher and closer to 1 the area under the curve (AUC) value is, the better the discriminatory power between the classes.

(16) $$\mathrm{TPR}=\frac{\;\mathrm{TP}\;}{\;\mathrm{TP}\;+\;\mathrm{FN}\;}$$

(17) $$\mathrm{FPR}=\frac{\;\mathrm{FP}\;}{\;\mathrm{FP}\;+\;\mathrm{TN}\;}$$

The AUC values are calculated for each classifier and each class. The performance of the decision tree is slightly worse than that of the other three classifiers for the four-wheeled and others classes. The random forest, the Gaussian SVM, and AdaBoost exhibit similar performance for the classes four-wheeled and others. A slight difference is observed for the random forest with the two-wheeled class.

## 7. Conclusions

This paper introduces a radar-based system for bike safety enhancement, leveraging coarse classification using an advanced two-level clustering technique to alert cyclists to potential dangers nearby. The system’s efficiency has been demonstrated through simulations and experiments involving both low- and high-resolution radar data. A dataset comprising synchronized radar point clouds and RGB camera images tailored to micromobility applications was collected. The effectiveness of the proposed clustering approach over the classical DBSCAN algorithm was validated.

Potential enhancements could entail expanding the training data to encompass a wider array of radar setups and environmental scenarios. Exploring automated data annotation techniques could accelerate the dataset’s consolidation. Adjusting radar and camera parameters during data capture might decrease noise and enhance the system’s performance. In addition, other ideas for future work include expanding a number of classes to distinguish between trucks, cars, and other classes.

This work establishes a proof of concept from the perspective of enhancing micromobility safety with radar sensing, illuminating a path for future breakthroughs in this domain.

## Figures and Tables

**Table 1:** Configuration of the radar sensor.

Parameter	Value
Maximum range	100 m
Range resolution	0.49 m
Maximum radial velocity	20.29 m/s
Radial velocity resolution	1.27 m/s
Azimuth resolution	14.5°
Number of chirps per frame	64
Number of samples per chirp	256
Frame rate	10 FPS
Sampling rate	12.5 Msps
Sweep bandwidth	307.2 MHz
Number of transmit antennas	2
Number of receive antennas	4

**Table 2:** Configuration of the camera sensor.

Parameter	Value
Frame resolution	640 × 480
Frame rate	25 FPS
FOV (H × V)	69° × 42°
sensor resolution	2 MP

**Table 3:** Hyperparameter selection—key metrics’ evolution vs. *N* and ϵ.

Key Metric	$\varepsilon_{2}$ for $N_{min_{2}}=2$												
	0.1	0.2	0.3	0.4	0.5	0.6	0.7	0.8	0.9	1.0	1.1	1.2	1.3
Homogeneity	0.488	0.737	0.850	0.891	0.936	0.955	0.955	0.902	0.903	0.908	0.908	0.906	0.906
Completeness	0.456	0.603	0.746	0.763	0.790	0.820	0.813	0.809	0.803	0.797	0.787	0.781	0.781
V-score	0.471	0.663	0.795	0.822	0.857	0.882	0.878	0.853	0.850	0.849	0.843	0.839	0.839
Key Metric	$\varepsilon_{2}$ for $N_{min_{2}}=3$												
	0.1	0.2	0.3	0.4	0.5	0.6	0.7	0.8	0.9	1	1.1	1.2	1.3
Homogeneity	0.318	0.618	0.784	0.849	0.903	0.921	**0.938**	0.885	0.885	0.891	0.891	0.889	0.889
Completeness	0.471	0.659	0.792	0.838	0.865	0.898	**0.889**	0.889	0.889	0.889	0.867	0.852	0.852
V-measure	0.380	0.639	0.789	0.844	0.883	0.909	**0.913**	0.887	0.887	0.889	0.879	0.869	0.869
Key Metric	$\varepsilon_{2}$ for $N_{min_{2}}=4$												
	0.1	0.2	0.3	0.4	0.5 e	0.6	0.7	0.8	0.9	1.0	1.1	1.2	1.3
Homogeneity	0.176	0.573	0.729	0.808	0.853	0.875	0.875	0.858	0.858	0.864	0.864	0.861	0.861
Completeness	0.450	0.687	0.787	0.851	0.890	0.943	0.943	0.928	0.928	0.912	0.912	0.907	0.907
V-score	0.253	0.625	0.757	0.829	0.871	0.908	0.908	0.892	0.892	0.887	0.887	0.883	0.883

**Table 4:** Statistics of different clustering metrics for the 2-level and DBSCAN algorithms.

Method		H	C	V	AR	DB	S
2-Level	Mean	0.91	0.91	0.91	0.86	0.16	0.53
	Min	0.79	0.78	0.87	0.72	0.09	0.12
	Max	0.98	0.97	0.98	0.99	0.21	0.87
DBSCAN	Mean	0.89	0.88	0.88	0.83	0.18	0.5
	Min	0.77	0.73	0.81	0.69	0.07	−0.2
	Max	0.96	0.95	0.97	0.97	0.33	0.86

**Table 5:** Scores for extracted features.

Feature	Score
dimension over x-axis	0.32
dimension over y-axis	0.24
mean velocity	0.21
equivalent RCS	0.19
density	0.11
standard deviation of RCS	0.09

**Table 6:** Classification results based on selected features using different classifiers.

Accuracy	Feature Set	Classifier			
		DT	RF	Gaussian SVM	AdaBoost
Validation	Proposed feature set	83.2%	87.8%	88.4%	86.6%
	Feature set from Ref. [17]	77.8 %	82.3%	81.6%	81.2%
Test	Proposed feature set	80.8%	85.9%	85.9%	86.4%
	Feature set from Ref. [17]	82.6%	86.1%	83.9%	84.8%

## Data Availability

Data sharing is not applicable.

## Abbreviations

The following abbreviations are used in this manuscript:

AR	Adjusted Rand
AUC	Area Under the Curve
C	Completeness
CNN	Convolutional Neural Network
DB	Davies–Bouldin Index
DBSCAN	Density-Based Spatial Clustering of Applications with Noise
DL	Deep Learning
DT	Decision Tree
ESPRIT	Estimation of Signal Parameters via Rotational Invariant Techniques
FFT	Fast Fourier Transform
FMCW	Frequency-Modulated Continuous Wave
FPR	False Positive Rate
FPS	Frame Rate per Second
GHz	Giga Hertz
H	Homogeneity
ITS	Intelligent Transportation Systems
MCE	Minimum Classification Error
MDPI	Multidisciplinary Digital Publishing Institute
MIQ	Mutual Information Quotient
ML	Machine Learning
MRMR	Minimum Redundancy–Maximum Relevance
Pedelec	Pedal Electric Cycles
RCS	Radar Cross Section
RF	Random Forest
RGB	Red Green Blue
ROC	Receiver Operating Characteristic
ROS	Robot Operating System
S	Silhouette
SVM	Support Vector Machine
US	United States
V	V-measure
VGG16	Very Deep Convolutional Networks with 16 layers
VoD	View of Delft
VRUs	Vulnerable Road Users
YOLO	You Only Look Once
TI	Texas Instruments
TPR	True Positive Rate

## References

[1] (2023). Report of the National Highway Traffic Safety Administration (NHTSA). Traffic Safety Facts: 2021 Data. https://crashstats.nhtsa.dot.gov/Api/Public/ViewPublication/813484.

[2] Kalaiselvan K. (2022). Early Warning System for Safe Lateral Maneuver of Bicycles. Master’s Thesis.

[3] Englund C., Clasen H., Bui T.H., Lindström D., Andersson J., Englund C., Clasen H., Lindström D. Radar system for bicycle—A new measure for safety. Proceedings of the ITS World Congress.

[4] Degen C., Domnik C., Kürten A., Meuleners M., Notz M., Pohle-Fröhlich R., Naroska E. Driver Assistance System for Pedelecs. Proceedings of the 2019 20th International Radar Symposium (IRS).

[5] Dorn C., Kurin T., Erhardt S., Lurz F., Hagelauer A. Signal Processing for Low-Power and Low-Cost Radar Systems in Bicycle Safety Applications. Proceedings of the 2022 IEEE Topical Conference on Wireless Sensors and Sensor Networks (WiSNeT).

[6] Hägelen M., Jetten R., Kassner J., Kulke R. Safety and Comfort Enhancement with Radar for a Bicycle Assistance System. Proceedings of the 2019 20th International Radar Symposium (IRS).

[7] Lang A.H., Vora S., Caesar H., Zhou L., Yang J., Beijbom O. PointPillars: Fast Encoders for Object Detection From Point Clouds. Proceedings of the IEEE/CVF Conference on Computer Vision and Pattern Recognition.

[8] Palffy A., Pool E., Baratam S., Kooij J.F., Gavrila D.M. (2022). Multi-class road user detection with 3+ 1D radar in the View-of-Delft dataset. IEEE Robot. Autom. Lett..

[9] Ester M., Kriegel H.P., Sander J., Xu X. A density-based algorithm for discovering clusters in large spatial databases with noise. Proceedings of the Second International Conference on Knowledge Discovery in Databases and Data Mining (KDD).

[10] Cortes C., Vapnik V. (1997). Support-vector networks. Mach. Learn..

[11] Kim S., Lee S., Doo S., Shim B. Moving Target Classification in Automotive Radar Systems Using Convolutional Recurrent Neural Networks. Proceedings of the 2018 26th European Signal Processing Conference (EUSIPCO).

[12] Yanik M.E., Rao S. Radar-Based Multiple Target Classification in Complex Environments Using 1D-CNN Models. Proceedings of the 2023 IEEE Radar Conference (RadarConf23).

[13] Kim W., Cho H., Kim J., Kim B., Lee S. (2020). YOLO-Based Simultaneous Target Detection and Classification in Automotive FMCW Radar Systems. Sensors.

[14] Lamane M., Tabaa M., Klilou A. (2023). New Approach Based on Pix2Pix–YOLOv7 mmWave Radar for Target Detection and Classification. Sensors.

[15] Palffy A., Dong J., Kooij J.F., Gavrila D.M. (2020). CNN Based Road User Detection Using the 3D Radar Cube. IEEE Robot. Autom. Lett..

[16] Gao X., Xing G., Roy S., Liu H. Experiments with mmWave Automotive Radar Test-bed. Proceedings of the 53rd Asilomar Conference on Signals, Systems, and Computers.

[17] Zhao Z., Song Y., Cui F., Zhu J., Song C., Xu Z., Ding K. (2020). Point Cloud Features-Based Kernel SVM for Human-Vehicle Classification in Millimeter Wave Radar. IEEE Access.

[18] Rizik A., Tavanti E., Vio R., Delucchi A., Chible H., Randazzo A., Caviglia D.D. Single Target Recognition Using a Low-Cost FMCW Radar Based on Spectrum Analysis. Proceedings of the 27th IEEE International Conference on Electronics, Circuits and Systems, (ICECS).

[19] Schubert E., Meinl F., Kunert M., Menzel W. Clustering of High Resolution Automotive Radar Detections and Subsequent Feature Extraction for Classification of Road Users. Proceedings of the 16th International Radar Symposium (IRS).

[20] Scheiner N., Appenrodt N., Dickmann J. Sick A Multi-Stage Clustering Framework for Automotive Radar Data. Proceedings of the 2019 IEEE Intelligent Transportation Systems Conference, (ITSC).

[21] Xie S., Wang C., Yang X., Wan Y., Zeng T., Liu Z. Millimeter-Wave Radar Target Detection Based on Inter-frame DBSCAN Clustering. Proceedings of the 22nd IEEE International Conference on Communication Technology (ICCT).

[22] Lu Y., Balachandran A., Tharmarasa R., Chomal S. Detection Level and Target Level Road User Classification with Radar Point Cloud. Proceedings of the 2023 IEEE Sensors Applications Symposium (SAS).

[23] Kim W., Cho H., Kim J., Kim B., Lee S. Target Classification Using Combined YOLO-SVM in High-Resolution Automotive FMCW Radar. Proceedings of the 2020 IEEE Radar Conference (RadarConf20).

[24] Gao T., Lai Z., Mei Z., Wu Q. Hybrid SVM-CNN Classification Technique for Moving Targets in Automotive FMCW Radar System. Proceedings of the 11th International Conference on Wireless Communications and Signal Processing (WCSP).

[25] Fang W.H., Fang L.D. (2020). Joint Angle and Range Estimation with Signal Clustering in FMCW Radar. IEEE Sens..

[26] Noureddine B.O., Walid R., Olivier J. (2022). Maneuvering Aid for cyclists. INPI Patent.

[27] Rosenberg A., Hirschberg J. V-Measure: A Conditional Entropy-Based External Cluster Evaluation Measure. Proceedings of the 2007 Joint Conference on Empirical Methods in Natural Language Processing and Computational Natural Language Learning (EMNLP-CoNLL).

[28] Warrens M.J., van der Hoef H. (2022). Understanding the adjusted rand index and other partition comparison indices based on counting object pairs. J. Classif..

[29] Davies D.L., Bouldin D.W. (1979). A cluster separation measure. IEEE Trans. Pattern Anal. Mach. Intell..

[30] Kaufman L., Rousseeuw P.J. (2009). Finding Groups in Data: An Introduction to Cluster Analysis.

[31] Ding C., Peng H. (2005). Minimum Redundancy Feature Selection from Microarray Gene Expression Data. J. Bioinform. Comput. Biol..

[32] Rokach L., Maimon O. (2005). Decision trees. Data Mining and Knowledge Discovery Handbook.

[33] Breiman L. (2001). Random Forests. Mach. Learn..

[34] Chengsheng T., Huacheng L., Bing X. (2017). AdaBoost typical Algorithm and its application research. MATEC Web Conf..

[35] Kohavi R. (1995). A Study of Cross-Validation and Bootstrap for Accuracy Estimation and Model Selection. IJCAI.

[36] Snoek J., Larochelle H., Adams R.P. (2012). Practical Bayesian Optimization of Machine Learning Algorithms. Advances in Neural Information Processing Systems.

[37] Matton M., Van Compernolle D., Cools R. (2010). Minimum classification error training in example based speech and pattern recognition using sparse weight matrices. Comput. Appl. Math..

[38] Bradley A.P. (1997). The use of the area under the ROC curve in the evaluation of machine learning algorithms. Pattern Recognit..

